# Supplementary material for: Clinician’s perspectives on gene therapy for Alzheimer’s disease: A qualitative study
Source: PLoS One. 2024 Jul 18;19(7):e0307567. doi: 10.1371/journal.pone.0307567 (PMC11257239; doi:10.1371/journal.pone.0307567)
Supplement: S1 File — (PDF) [file pone.0307567.s001.pdf]

Campus IRB: (2022-0149) - [Protocol Approval]

Campus IRB <campusirb@duke.edu>
Tue 12/21/2021 2:58 PM
To:Nathan Boucher <nathan.boucher@duke.edu>

Protocol: 2022-0149
Exploring Gene Therapy Understanding and Values in Patients with Dementia, their Caregivers, and Clinicians

Researcher(s):
Boucher, Nathan - Researcher
Yavarow, Zollie - Graduate student researcher
Keleman, Lilly - Undergraduate researcher
Gupta, Ishika - Undergraduate researcher
keith, Belinda - Manager

Check-In Date: 12/20/2022

The researchers listed on this protocol agree to:

1. Conduct the research in accordance with the approved protocol.
2. Secure approval before making any changes to the protocol, such as adding a new source of funding, adding a subject population, revising procedures, modifying the informed consent process, or replacing or adding investigators.
3. Renew the protocol within thirty days prior to the check-in date noted above.
4. Report any unanticipated risks to the research subjects or deviations from the procedures described in the protocol to the IRB Staff as soon as they are identified.
5. Notify the IRB Staff when the research is completed.
6. Retain your original research data and signed consent forms for at least five years, in accordance with Duke's Policy on Research Records, Sharing, Retention, and Ownership. (Faculty Handbook, Appendix P)

Links to useful content on the IRB website:

- Subject Complaints, Setbacks and Other Problems & Issues guide
- [campusirb.duke.edu/node/22]Request to Amend an Approved Protocol form
- Periodic Check-In Form form

All consent processes should include the Protocol ID (2022-0149). Including the Protocol ID in each consent process will help participants identify the study in case they need to contact the research team or IRB. If your consent processes do not include the Protocol ID#, please add them at this time.
Adding the Protocol ID# to consents does not require an amendment.

===========

Dear Researchers,

Please review the Campus IRB's COVID-19 Updates page, which provides information on current research restrictions, return to research procedures, and where to learn more about research-releated updates regarding Duke's response to coronavirus (COVID-19).

Sincerely,

The Campus IRB
