# Supplementary material for: Clinician’s perspectives on gene therapy for Alzheimer’s disease: A qualitative study
Source: PLoS One. 2024 Jul 18;19(7):e0307567. doi: 10.1371/journal.pone.0307567 (PMC11257239; doi:10.1371/journal.pone.0307567)
Supplement: S2 File — (DOCX) [file pone.0307567.s002.docx]

**Semi-Structured Interview Guide for health care providers**

Thank you for agreeing to speak with me today. We are researchers from Duke seeking your thoughts on new Alzheimer’s treatment technologies. The questions I would like to ask deal with your experience with treating persons with dementia. I hope that understanding your experiences will help us to ­­­­­­­­­­­­­­­­understand the values of persons with dementia and their caregivers as new treatments are developed.

Your participation in this study is strictly confidential. The interview will be audio-recorded, and this simply provides for accurately keeping track of information. Subsequently the tape will be destroyed. Any names or identifying information mentioned during the interview will be redacted from the transcript. We would prefer to benefit from your input on all of these questions, but if you do not want to answer a question please let me know and we can skip to another question. Your participation in this study is important; however, should you at any time wish to stop, you may do so without penalty to you. At any time, please feel free to ask me questions concerning the interview or the study.

I will begin the recording by saying your unique Participant ID and then we will begin the interview. Do you have any questions before I being the recording? May we begin?

I am turning on the recording

[RESEARCHER STATES UNIQUE PARTICIPANT ID]

**DOMAIN: Establishing Understanding**

1. What level of understanding do your patients and their caregivers have about gene therapy?
   1. Follow with a standard explanation of gene therapy…
      1. *Gene therapy is an experimental technique that uses genes - a unit of heredity written in our DNA - to treat or prevent disease. In the future, this technique may allow health care providers to treat a disorder, such as Alzheimer’s disease by adding or changing a gene in a patient's cells.*
2. What do you think would be helpful for them to know about gene therapy?

**DOMAIN: Cost and Access**

1. Where do you get information about new Alzheimer’s care and treatment strategies?

What are some things you like about those information sources?

1. What would you consider before offering a newly approved Alzheimer’s treatment?

**DOMAIN: Quality of Life**

1. If a treatment is able to stop Alzheimer’s, how late would you recommend this type of treatment to a person with Alzheimer’s? Describe the stage of Alzheimer’s in your own words.
   1. PROBE: For example, what do symptoms have to look like before you question if a therapy is still beneficial?
   2. PROBE: [If they indicate it is always beneficial] Are you saying that this treatment should be given no matter the disease state?
2. According to the Alzheimer’s Association, Black Americans are diagnosed with Alzheimer’s at a later stage than White Americans.
   1. Why do you think this happens?
   2. What do you think should be done differently?

**DOMAIN: Personal Morals**

1. What level of risk is acceptable for an Alzheimer’s treatment? For example, what types of side effects or medical procedures should a patient tolerate?
2. Do you think patients would be more or less willing to accept risks when participating in a clinical trial? Please discuss.
3. What role does religion or spirituality play in your patients’ medical decisions?

a. Probe: To what degree do they discuss this type of thing with you?

1. At this time, do you have anything else to add?

That’s all the questions we have for you. Thank you for your time.

I am shutting off the recording.

Thank you again. Your opinions are valuable, and we appreciate your time.
