# Supplementary material for: Clinician’s perspectives on gene therapy for Alzheimer’s disease: A qualitative study
Source: PLoS One. 2024 Jul 18;19(7):e0307567. doi: 10.1371/journal.pone.0307567 (PMC11257239; doi:10.1371/journal.pone.0307567)
Supplement: Attachment — Submitted filename: Reviewer 2 Response.docx [file pone.0307567.s004.docx]

Reviewer #2: Dear authors,

Although most of my comments have been addressed appropriately, I still have one major issue:

The introduction has been rewritten and expanded to clarify that this paper addresses clinician's views on gene therapies targeted at the APOE4 allele.

However the methods section now states that participants were provided the following - very general - definition of gene therapies: "Gene therapy is an experimental technique that uses genes - a unit of heredity written in our DNA - to treat or prevent disease. In the future, this technique may allow health care providers to treat a disorder, such as Alzheimer’s disease by adding or changing a gene in a patient's cells."

Yet ApoE4 is not mentioned anywhere in the interview guide. Moreover, most questions, except for the first one, relate to "new Alzheimer’s care and treatment strategies", or "a treatment is able to stop Alzheimer’s" or "an Alzheimer’s treatment". Some do not even mention treatment of any kind ("6. According to the Alzheimer’s Association, Black Americans are diagnosed with Alzheimer’s at a later stage than White Americans. a. Why do you think this happens?"). The interview guide does not seem to focus on gene therapies targeted at ApoE4, or even gene therapies in general, but at any kind of hypothetical new strategy for treatment or care.

I think this is problematic, as the clinicians who were interviewed, seemed to talk about treatments in a very broad sense, given their comments about patients' decision-making capacities, assuming they are cognitively impaired, even confined to bed, or have “progressed to such a degree that it seems that any further treatment would just be burdensome”. In my opinion it is extremely unlikely that gene therapies would be offered to patients with MCI or dementia. So I get the impression that the results do not relate to gene therapies targeted at ApoE4, but to any kind of treatment or care in any stage of the disease.

Thank you for these additional comments. We changed the final paragraph of the introduction and the first paragraph of the discussion to address the issue of addressing views specifically on gene therapy with APOE4 which as you point out is in error. We agreed with your comment. We changed the language to mention the possible gene therapies with AD, but to state that the study addressed general gene therapy for AD. We agree that we assess views on novel treatments, but this is in the context of gene therapy as a novel treatment. We feel it is important to guage clinician decision making of novel treatments in the MCI/dementia due to AD population. Many novel therapies for AD, including gene therapy will most likely be tested or trialed in a cognitively impaired population before testing in a cognitively normal population, for safety reasons. We state this in the conclusion. We thank you for your review and opportunity to improve our paper on these important ideas related to possible future AD treatments.
